# Retrograde Duodenojejunal Intussusception Caused by Ladd's Bands in an Adult With Intestinal Malrotation: A Rare Variant of Waugh's Syndrome

**DOI:** 10.7759/cureus.104279

**Published:** 2026-02-26

**Authors:** Tatsuya Koyama, Hiroyuki Kobayashi, Satoshi Kaihara

**Affiliations:** 1 Department of Surgery, Kobe City Medical Center General Hospital, Kobe, JPN

**Keywords:** intussusception, ladd’s bands, ladd's procedure, malrotation, retrograde intussusception, waugh’s syndrome

## Abstract

We report a rare case of retrograde duodenojejunal intussusception caused by Ladd's bands in an adult with intestinal malrotation. A 27-year-old man with a history of cerebral palsy and gastrostomy presented with frequent vomiting. Abdominal contrast-enhanced computed tomography showed marked distention of the stomach and duodenum, along with retrograde intussusception of the jejunum into the duodenum at the horizontal segment. No neoplastic lesions were identified as a lead point. A diagnosis of intestinal obstruction due to retrograde intussusception was made, and emergency laparoscopic surgery was performed.

Intraoperative findings revealed nonrotation-type intestinal malrotation. The duodenum was not fixed to the retroperitoneum, and a thickened Ladd's band was pulling the duodenum ventrally. This mechanical traction caused the mobile jejunum to invaginate retrogradely into the duodenum. Transection of the Ladd's band released the traction, resulting in spontaneous reduction of the intussusception. No ischemic changes were observed in the intestine, and the postoperative course was uneventful.

Retrograde intussusception in adults is extremely rare and is typically caused by organic lesions or postoperative factors. In this case, mechanical traction by Ladd's bands combined with intestinal mobility due to malrotation was considered the primary cause. Laparoscopic surgery was effective for both definitive diagnosis and minimally invasive treatment.

## Introduction

The association of malrotation and intussusception is known as Waugh’s syndrome. Intestinal malrotation is a congenital anomaly resulting from the failure of midgut rotation and fixation during embryonic development. The incidence of malrotation varies from 1:200 to 1:6,000 live births [1], with 90% of cases being diagnosed in the first year of life [2]. The incidence in the adult population is 0.2% [3].

Conversely, retrograde intussusception-where the distal segment of the intestine invaginates into the proximal segment-is an exceptionally rare condition, accounting for only 0.6%-1.9% of all adult intussusceptions [4]. Here, we report an extremely rare case of retrograde duodenal intussusception caused by Ladd's bands in an adult with intestinal malrotation. Ladd's bands are abnormal fibrous stalks of peritoneal tissue typically associated with this anomaly. The patient was successfully treated via laparoscopic surgery.

## Case presentation

The patient is a 27-year-old man with a complex medical history including cerebral palsy, epilepsy, tracheomalacia (managed via tracheostomy and ventilator), gastrostomy, and a prior brachiocephalic artery transection. In 2025, he began experiencing frequent vomiting, leading to a referral to our hospital after an abdominal computed tomography (CT) by his primary physician suggested severe gastric dilatation and intestinal obstruction.

**Figure 1:**
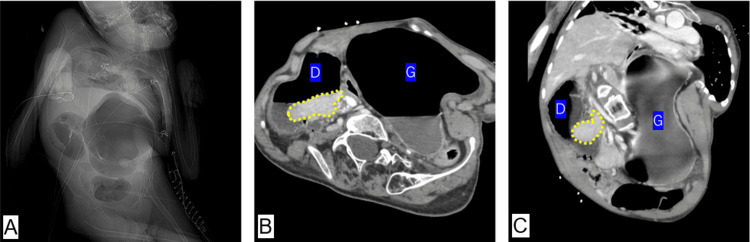
Contrast-enhanced abdominal CT ((A) scout, (B) axial, and (C) coronal views): severe scoliosis is noted, along with marked gastroduodenal distention and retrograde intussusception of the jejunum (outlined in yellow) into the horizontal segment of the duodenum. G: gastric; D: duodenum; CT: computed tomography

Upon admission, physical examination showed a height of 134 cm and a weight of 24.2 kg with stable vital signs; while the abdomen was slightly distended, it remained soft without tenderness or signs of peritoneal irritation. Laboratory findings were largely unremarkable, save for a slightly elevated inflammatory response (WBC: 18,300/μL, normal range: 3,500-9,000/μL; CRP: 0.82 mg/dL, normal range: <0.30 mg/dL). Imaging via contrast-enhanced CT revealed severe scoliosis and marked distention from the stomach to the duodenum. Specifically, at the horizontal segment of the duodenum, a retrograde intussusception-where the distal jejunum had invaginated into the proximal duodenum-was identified as the cause of the obstruction, with no obvious neoplastic lead point (Figure 1).

**Figure 2:**
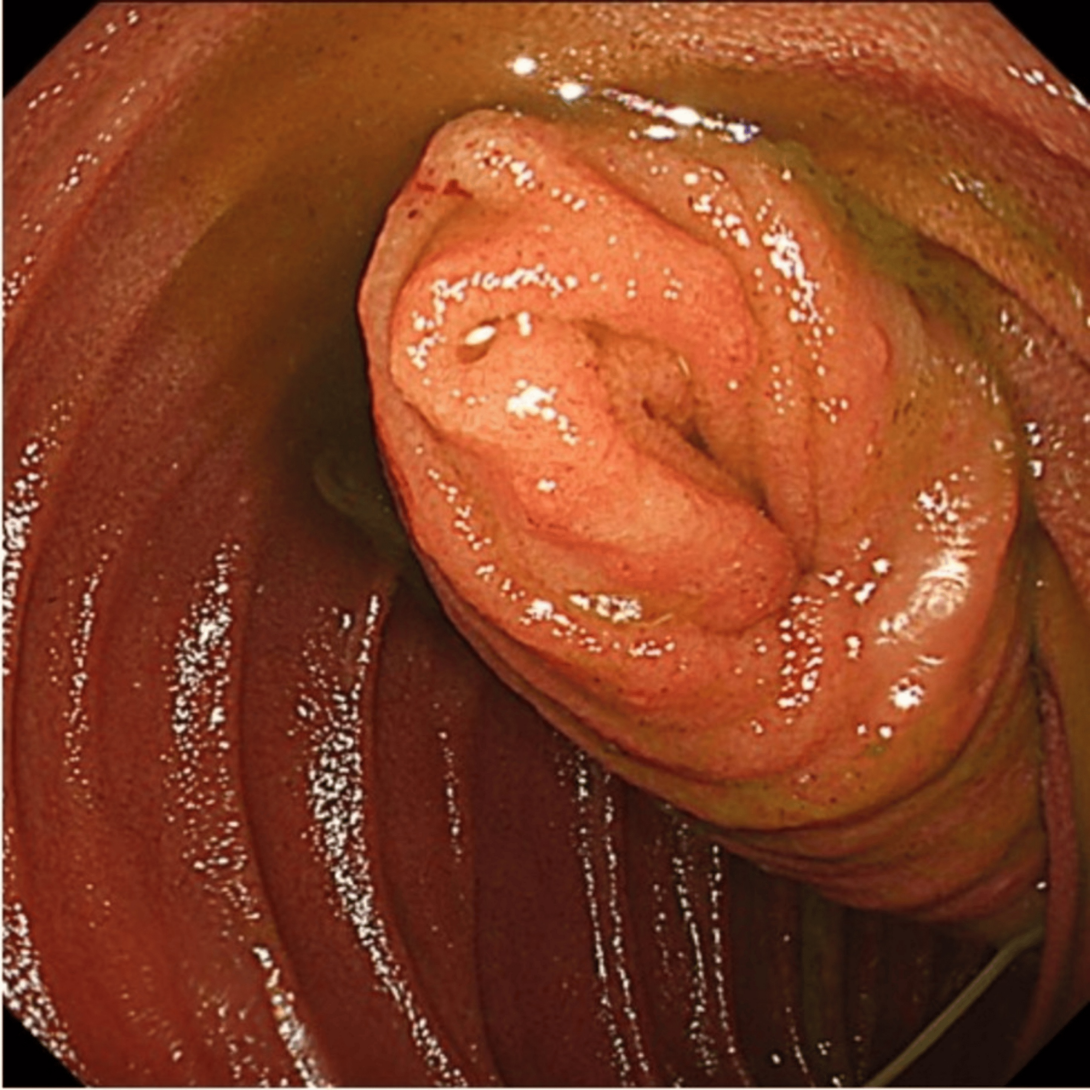
Esophagogastroduodenoscopy (EGD) demonstrated that the distal intestine was invaginated retrogradely into the duodenum, with no evidence of a neoplastic lead point.

Although an initial attempt at endoscopic reduction confirmed the invagination, it failed to achieve reduction, and no tumorous lesions were visualized (Figure 2).

Consequently, the patient underwent emergency laparoscopic surgery under a preoperative diagnosis of intestinal obstruction. Intraoperative findings confirmed nonrotation-type intestinal malrotation, characterized by the small intestine positioned on the right and the large intestine on the left. The duodenum lacked retroperitoneal fixation, and the ligament of Treitz was absent. Notably, a thickened Ladd's band extending from the duodenum toward the right abdominal wall exerted strong ventral traction, which had triggered the retrograde invagination of the distal jejunum.

**Figure 3:**
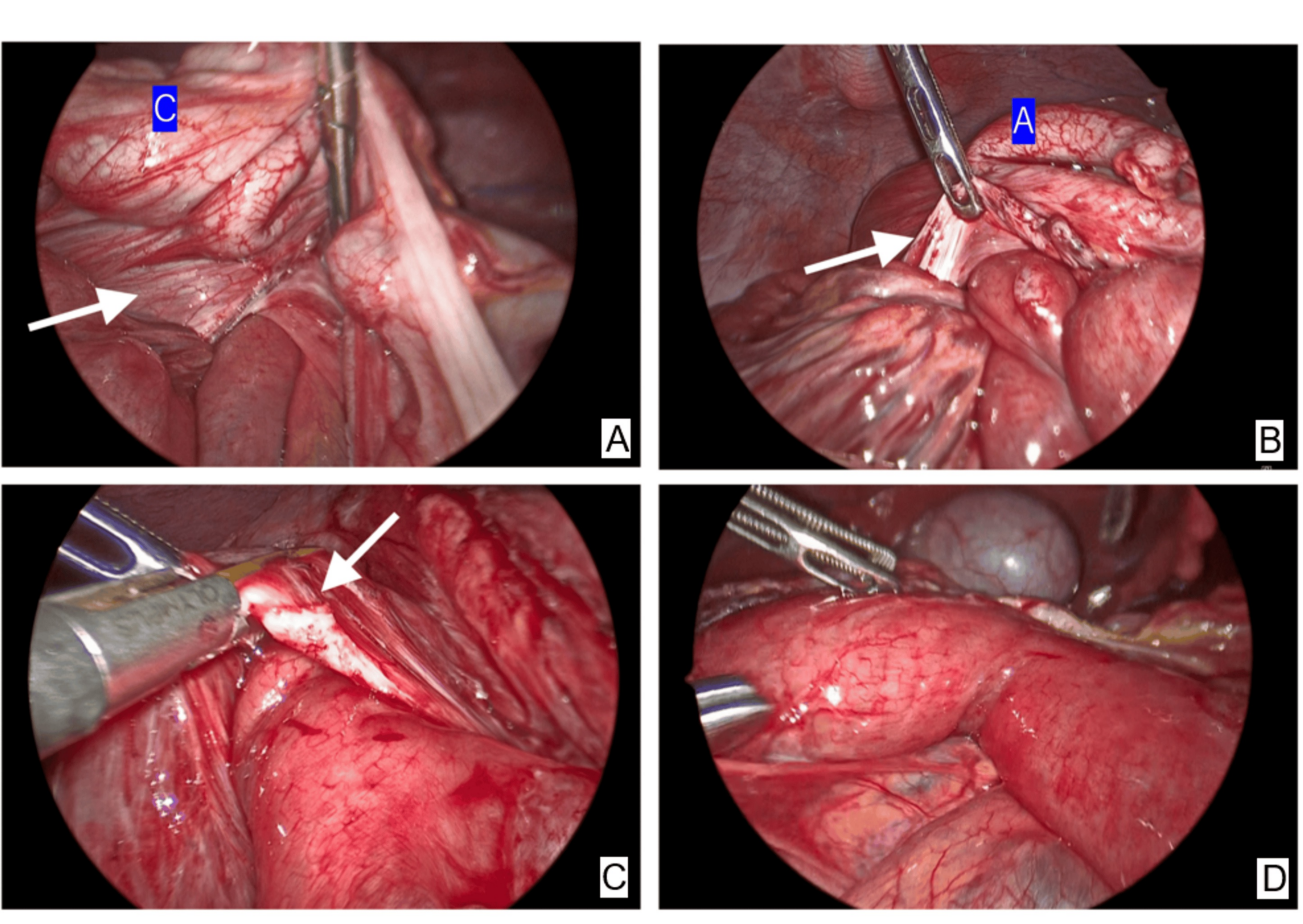
(A-D) Laparoscopic findings: the duodenum was tethered posteriorly by a thickened Ladd’s band (white arrow), with the distal jejunum invaginated retrogradely into the proximal segment. The duodenal intussusception was reduced following the transection of the Ladd’s band. C: cecum; A: appendix

The surgical procedure involved transecting the Ladd's band with an ultrasonically activated device, which released the traction and allowed for the spontaneous reduction of the intussusception (Figure 3).

No signs of ischemia or necrosis were observed. Following intraoperative endoscopic confirmation of full reduction and adequate passage, the procedure was completed. The postoperative course was stable; although delayed gastric emptying necessitated temporary total parenteral nutrition, enteral nutrition via gastrostomy was successfully resumed by day 22. The patient experienced no further vomiting and was discharged on postoperative day 40.

## Discussion

Intestinal malrotation is defined as an arrest in the normal 270° counter-clockwise rotation of the midgut around the superior mesenteric artery (SMA) during early embryonic development [5]. According to Wang’s classification [6], this case was a "nonrotation type," where the lack of retroperitoneal fixation of the duodenum provided the anatomical basis for the pathology. The Ladd procedure is necessary for intestinal malrotation, which includes dividing the Ladd's bands, mobilizing the duodenum and right colon, adhesiolysis near the superior mesenteric vessels, and appendectomy. The routine use of cecopexy is controversial [1]. In this case, an appendectomy was not performed.

The association between intestinal malrotation and intussusception was first described by Waugh in 1911 [7] and subsequently named "Waugh's syndrome" by Brereton et al. in 1982 [8]. The standard treatment remains the Ladd procedure.

To identify relevant literature on the topic, a systematic search was carried out in PubMed. A combination of "malrotation" and "intussusception" and "adult" keywords was used during the search. A total of nine case reports were identified in adults. The available literature is presented in Table 1.

**Table 1:** A summary of adult case reports involving both intestinal malrotation and intussusception.

Author and year	Age/gender	Lead point	Direction of intussusception	Procedure
Gardner-Thorpe et al. (2007) [9]	66/female	Ampullary villous adenoma	Antegrade (duodeno-duodenal)	Laparotomy (pancreatoduodenectomy)
Hsieh et al. (2008) [10]	56/female	Inflammatory fibroid polyp	Antegrade (ileo-ileal)	Conservative treatment and later managed with laparotomy. The Ladd procedure was also applied
Bayan et al. (2009) [11]	34/female	Brunner's gland hamartoma	Antegrade (duodeno-jejunal)	Laparotomy (after longitudinal duodenotomy, a polyp was excised)
Chaudhary et al. (2012) [12]	25/female	None	Antegrade (ileo-colic)	Laparotomy (resection of distal ileum, cecum, and proximal half of ascending colon)
Gandhi et al. (2019) [13]	60/female	Villous adenoma	Antegrade (colo-colic)	Laparotomy (segmental transverse colectomy and appendectomy)
Santos-Seoane et al. (2019) [14]	24/female	None	Antegrade (ileo-ileal)	Conservative treatment
Sousa and Ferreira (2020) [15]	56/female	None	Antegrade (ileo-colic)	Laparotomy (right hemicolectomy)
Afasha et al. (2024) [16]	19/male	None	Antegrade (ileo-colic)	Laparotomy (colectomy)
Shenoy et al. (2024) [17]	27/female	Tubulovillous adenoma	Antegrade (duodeno-duodenal)	Laparotomy (anterior duodenotomy)

In summary, intussusception was antegrade in all cases. A lead point was absent in four cases, and all except one underwent surgical intervention.

Retrograde intussusception, where the distal segment of the intestine invaginates into the proximal segment, is an exceptionally rare condition, representing only 0.6% to 1.9% of all adult intussusceptions [4]. It is most frequently associated with prior gastric surgery (particularly Braun anastomosis), intraluminal tumors, or iatrogenic factors such as the removal of an ileus tube. Although our patient had a history of gastrostomy, preoperative endoscopy and intraoperative findings showed no organic lesions or localized wall thickening at the gastrostomy site, allowing us to rule it out as a "lead point."

We hypothesize that three distinct anatomical factors converged to cause the retrograde duodenal intussusception in this patient. First, the nonrotation malrotation meant the duodenum and proximal jejunum lacked retroperitoneal fixation, providing the necessary mobility for invagination. Second, the thickened Ladd's band exerted strong mechanical traction, pulling the duodenum ventrally and toward the right abdominal wall. This lateral and ventral displacement likely created a "funnel" effect that facilitated the retrograde entry of the mobile jejunum into the duodenal lumen. Third, the patient’s severe scoliosis significantly constrained and deviated the intra-abdominal space, potentially forcing the mobile jejunal loops into the fixed-yet-tethered duodenal segment against the normal direction of peristalsis.

Laparoscopic surgery proved highly valuable for both diagnosis and treatment. Crucially, while no clear lead point was identified in preoperative imaging or endoscopy, the ability to directly visualize the dynamic pathology-traction of the duodenum by the Ladd's band-and achieve cure with minimal necessary manipulation (band transection) highlights its significance as both a diagnostic and minimally invasive approach.

## Conclusions

We encountered an extremely rare case of adult intestinal malrotation presenting as retrograde duodenal intussusception caused by Ladd's bands. This condition should be considered a potential cause of unexplained intestinal obstruction. Laparoscopic surgery proved to be an invaluable tool for both definitive diagnosis and effective treatment.
